# Plant Adaptation to Multiple Stresses during Submergence and Following Desubmergence

**DOI:** 10.3390/ijms161226226

**Published:** 2015-12-17

**Authors:** Bishal Gole Tamang, Takeshi Fukao

**Affiliations:** 1 Department of Crop and Soil Environmental Sciences, Virginia Tech, Blacksburg, VA 24061, USA; bgtamang@vt.edu; 2 Translational Plant Sciences Program, Virginia Tech, Blacksburg, VA 24061, USA; 3 Fralin Life Science Institute, Virginia Tech, Blacksburg, VA 24061, USA

**Keywords:** flooding, oxidative stress, dehydration, starvation, salinity, disease

## Abstract

Plants require water for growth and development, but excessive water negatively affects their productivity and viability. Flash floods occasionally result in complete submergence of plants in agricultural and natural ecosystems. When immersed in water, plants encounter multiple stresses including low oxygen, low light, nutrient deficiency, and high risk of infection. As floodwaters subside, submerged plants are abruptly exposed to higher oxygen concentration and greater light intensity, which can induce post-submergence injury caused by oxidative stress, high light, and dehydration. Recent studies have emphasized the significance of multiple stress tolerance in the survival of submergence and prompt recovery following desubmergence. A mechanistic understanding of acclimation responses to submergence at molecular and physiological levels can contribute to the deciphering of the regulatory networks governing tolerance to other environmental stresses that occur simultaneously or sequentially in the natural progress of a flood event.

## 1. Introduction

Over the past six decades, flooding events have increasingly occurred throughout the world as a consequence of global climate change [1]. Flooding is a major natural disaster that has a detrimental effect on plant growth and fitness in natural and agricultural ecosystems [2]. Although prolonged flooding substantially impacts their productivity and viability, plants are equipped with the acclimation mechanisms to cope with a transient influx of water into their environment. Such adaptive responses include energy generation through fermentative metabolism in the absence of oxygen, development of aerenchyma and adventitious roots for improved aeration, a reduction in cuticle and epidermal cell wall thickness for decreased diffusion resistance, activation of internode and petiole elongation to outgrow submergence water, and restriction of growth for the conservation of precious energy until floodwater subsides [3,4]. These species-specific or common responses to flooding allow plants to endure or avoid excess water, conferring enhanced adaptation and survival under the stress.

**Figure 1:**
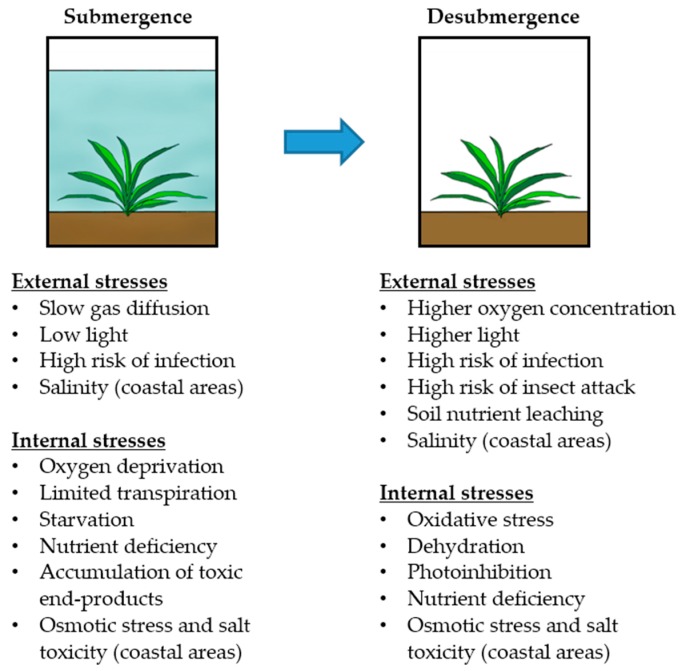
External and internal stresses induced during submergence and following desubmergence in plants. When immersed in water, plants encounter drastic changes in environmental parameters (external stresses), triggering a variety of internal stresses. When floodwaters recede, submerged plants are suddenly exposed to aerobic conditions, inducing additional external and internal challenges. To overcome submergence and post-submergence stresses, plants require tolerance to multiple stresses that occur simultaneously or sequentially over a flood event.

Submergence is a type of flooding stress and is defined as a condition where the entire plant is fully immersed in water (complete submergence) or at least part of the shoot terminal is maintained above the water surface (partial submergence). This review mainly focuses on plant responses to complete submergence and its associated stresses at the molecular and physiological levels. Under submergence and subsequent desubmergence, plants face multiple external challenges simultaneously or sequentially, which generate various internal stresses that affect plant growth and survival (Figure 1). Submergence substantially decreases the rate of gas diffusion, limiting oxygen uptake and compelling carbon inefficient anaerobic metabolism [5]. Turbid floodwaters reduce light availability, inhibiting underwater photosynthesis. Limitation of efficient gas exchange also restricts transpiration severely [6], possibly impeding the absorption and transport of nutrients from the soil. Under prolonged submergence, these conditions induce energy starvation and nutrient deficiency in plants. Continuous anaerobic metabolism can result in the accumulation of phytotoxic end-products [3]. When floodwaters subside, submerged plants encounter the rapid entry of oxygen, causing oxidative damage through overproduction of reactive oxygen species (ROS) and toxic oxidative products [7,8]. Likewise, sudden exposure to higher light can induce photooxidative damage to photosystem II reaction centers, leading to reduced photosynthetic capacity (photoinhibition) [9]. Desiccation of leaves following desubmergence is also observed due to a marked reduction in hydraulic conductivity in shoots [10]. Nutrient deficiency can persist after desubmergence because of mineral leaching from the soil. Submergence and post-submergence stresses can increase the probability of pathogen infection since high humidity and heavy rainfall favor pathogen development and disease transmission [11,12]. It has been shown that submergence attenuates plant resistance to insect herbivores [13,14], which raises the risk of insect damage upon desubmergence. In low-lying lands of coastal regions, plants can be submerged in seawater as a result of high tides, storm surges, and tsunami. Inundation of seawater can lead to salinization of arable soils, which may last for long periods of time after flooding. From the above, it is obvious that plants suffer from multiple external and internal stresses during the natural progression of a flood event. In this review, we discuss how plants coordinate multiple adaptation mechanisms to cope with various stresses that occur concurrently or subsequently during submergence and following desubmergence.

## 2. Molecular Mechanisms of Submergence Tolerance and Escape

A mechanistic understanding of molecular regulation underlying submergence tolerance and escape in plants has been advanced through functional characterization of key genes responsible for acclimation to these stresses in rice (*Oryza sativa*). Rice is a wetland plant that is well adapted to partially flooded conditions (root waterlogging). However, most rice accessions die when completely immersed in water for 7–10 days [15]. A limited number of rice varieties can tolerate a deep transient flash flood through economization of energy reserves (quiescence strategy) or escape from a slow progressive flood through rapid internode elongation (escape strategy) [1,16]. Quantitative trait locus (QTL) analysis and map-based cloning revealed that the *SUBMERGENCE1* (*SUB1*) locus, encoding a variable cluster of two or three tandem-repeated group VII of ETHYLENE RESPONSIVE FACTOR (ERF-VII), regulate the quiescence response [15]. All rice accessions surveyed contained *SUB1B* and *SUB1C* genes at the *SUB1* locus, whereas *SUB1A* was limited to some *indica* and *aus* varieties [15,17]. Conditional and constitutive expression of *SUB1A* conferred survival of complete submergence for 14–16 days [17,18,19]. Remarkably, the submergence escape response, contrasting with the quiescence response, is also primarily regulated by the locus containing tandem-repeated *ERF-VII* genes, designated *SNORKEL1* (*SK1*) and *SNORKEL2* (*SK2*) [20]. Allelic surveys revealed that *SK* genes are present only in deepwater rice accessions that exhibit rapid internode elongation in response to submergence.

**Figure 2:**
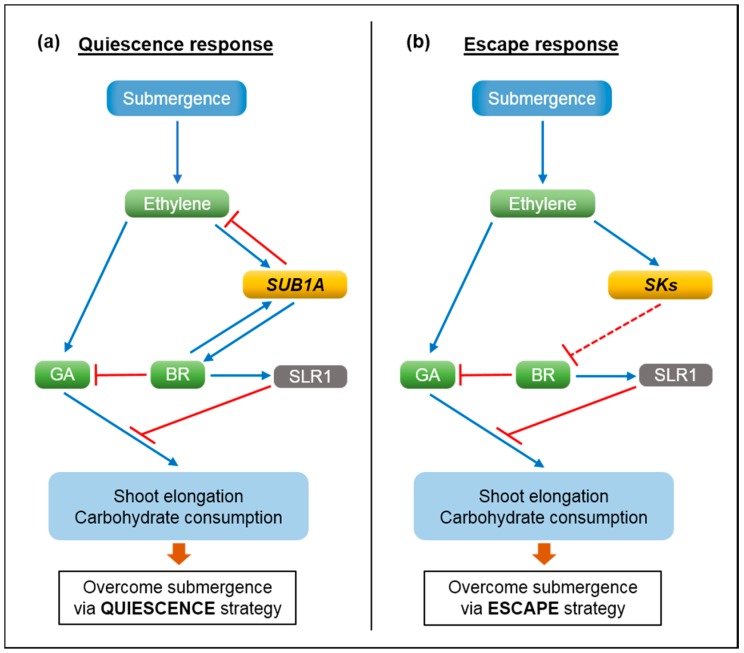
Model of the regulatory mechanisms underlying the quiescence and escape responses to submergence in rice. (**a**) Quiescence response: Under submergence, the level of endogenous ethylene quickly rises due to physical entrapment and increased biosynthesis, triggering mRNA accumulation of *SUB1A* [18]. *SUB1A* ultimately limits ethylene production, contributing to a reduction in ethylene-mediated GA biosynthesis. *SUB1A* also upregulates production of brassinosteroids (BR), promoting degradation of bioactive gibberellins (GA) and accumulation of SLR1, a negative regulator of GA signaling [21]. As a result, GA-mediated shoot elongation and carbohydrate consumption are suppressed in a *SUB1A*-dependent manner, enabling the avoidance of carbohydrate starvation and an energy crisis during submergence; (**b**) Escape response: Submergence-induced ethylene also increases the abundance of *SNORKEL* (*SK*) mRNAs [20]. It is anticipated that the regulatory role of BR in breakdown of bioactive GA and accumulation of SLR1 is conserved within *O. sativa* varieties. Based on the antithetical functions of *SUB1A* and *SKs*, upregulation of GA biosynthesis and responsiveness observed in deepwater rice [20,22,23] might be regulated via suppression of BR accumulation by *SKs*. This response allows deepwater rice to outgrow submergence water through GA-mediated internode elongation. Blue and red lines represent positive and negative regulation, respectively. A dashed line indicates a hypothetical relationship.

We propose that *SUB1A* and *SK* genes differentially regulate the hormonal network conserved in rice, modulating the two antithetical responses to submergence, respectively (Figure 2). Submergence promotes biosynthesis and entrapment of ethylene, which stimulates mRNA accumulation of *SUB1A* [18]. However, *SUB1A* ultimately limits ethylene production, leading to the suppression of ethylene-mediated production of gibberellic acids (GA). *SUB1A* also increases the abundance of brassinosteroids (BR), which enhances degradation of bioactive GA [21]. Increased BR levels also contribute to the accumulation of SLENDER RICE1 (SLR1), a DELLA protein that negatively regulates GA signaling. Positive feedback regulation of *SUB1A* and BR can further augment *SUB1A*-dependent hormonal regulation, resulting in the restriction of GA-mediated elongation growth and carbohydrate consumption under submergence (quiescence response). Similar to *SUB1A*, the abundance of *SK* transcripts is elevated by submergence-induced ethylene [20]. *SKs* promote accumulation of bioactive GA in submerged internodes [22]. It has been recognized that ethylene increases biosynthesis of and responsiveness to GA under submergence, triggering internode elongation in deepwater rice [23,24]. However, Hattori *et al.* [20] had demonstrated that *SKs* are not involved in ethylene production during submergence. As observed in other hormonal regulation, it is expected that the regulatory roles of BR in degradation of bioactive GA and accumulation of SLR1 are conserved within the same species (*i.e.*, *O. sativa*). Based on the contrasting roles of *SUB1A* and *SKs* in submergence responses, *SKs* could downregulate BR synthesis in deepwater rice, promoting GA-mediated elongation growth caused by increased production and signaling of GA.

The *Arabidopsis* (*Arabidopsis thaliana*) genome encodes five *ERF-VII* genes; two *HYPOXIA RESPONSIVE ERF* (*HRE*) genes, *HRE1* (*ERF73*) and *HRE2* (*ERF71*), and three *RELATED TO AP2* (*RAP2*) genes, *RAP2.2* (*ERF75*), *RAP2.3* (*ERF72*/*EBP*), and *RAP2.12* (*ERF74*) [1,25]. Investigation of overexpression lines and loss-of-function mutants has demonstrated that all *Arabidopsis ERF-VII*s are involved in adaptation to submergence and oxygen deprivation. Both hypoxia and anoxia dramatically increased the abundance of *HRE1* and *HRE2* mRNAs [26,27]. On the other hand, *RAP2.2*, *RAP2.3*, and *RAP2.12* mRNAs were accumulated even under normoxia in association with polysomes, suggesting that these proteins are constitutively synthesized [1,28]. Constitutive expression of each of these genes enhanced induction of the core hypoxia-responsive genes under oxygen deprivation, which were positively correlated with survival of seedlings under hypoxia and adult plants under submergence [26,27,29,30,31,32]. Consistently, a double-knockout mutant of *HRE1* and *HRE2* (*hre1hre2*) and single knockout mutants of *RAP2* genes (*rap2.2*, *rap2.3*, and *rap2.12*) exhibited reduced tolerance to hypoxia or submergence [27,29]. A recent study has revealed that *RAP2.12* promotes expression of a hypoxia-inducible trihelix transcription factor gene, *HYPOXIA RESPONSE ATTENUATOR 1* (*HRA1*), but HRA1 protein physically interacts with RAP2.12 protein to restrict its transactivation capacity [33]. Interestingly, HRA1 also downregulated activation of its own promoter. It was proposed in Giuntoli *et al.* [33] that these two feedback loops contribute to the fine-tuning of RAP2.12-mediated gene expression, providing adequate consumption of energy reserves during oxygen deprivation and submergence.

**Figure 3:**
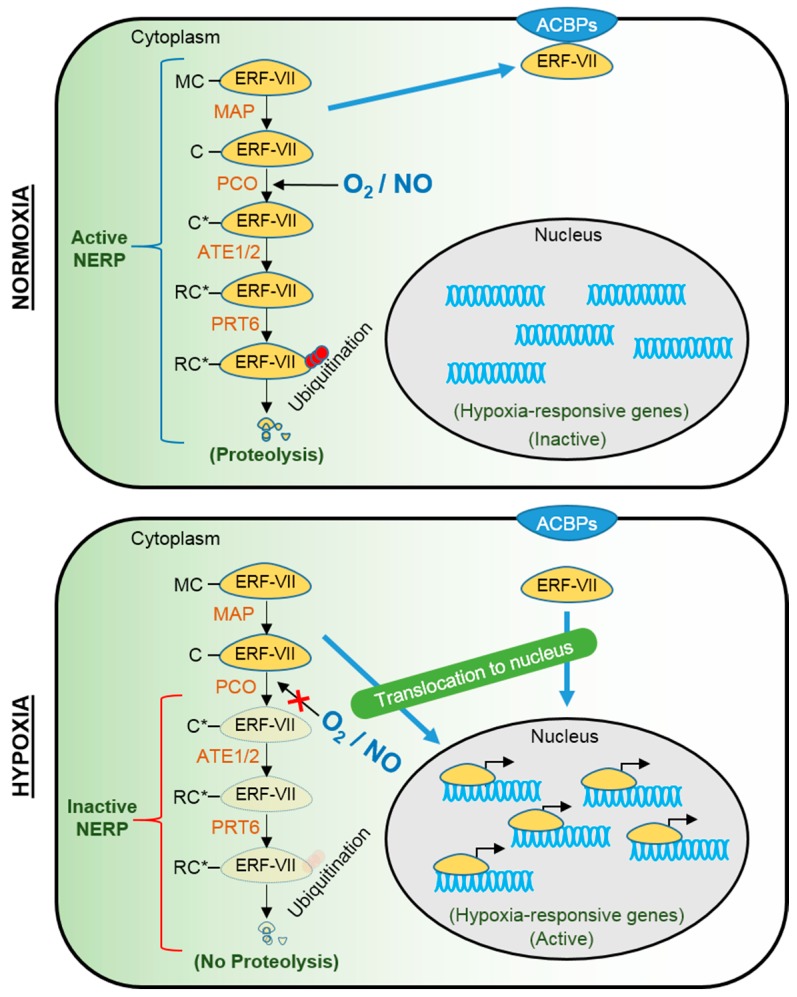
Oxygen-dependent stabilization and localization of ERF-VII proteins. Under oxygen-replete conditions (normoxia), ERF-VII proteins are degraded via the N-end rule pathway of proteolysis (NERP). All ERF-VII proteins contain methionine and cysteine (MC) at the N-terminal [25] and the first methionine (M) is constitutively cleaved by methionine aminopeptidase (MAP) [36]. The exposed cysteine (C) is converted to Cys-sulfinic or Cys-sulfonic acid (C*) by plant cysteine oxidase (PCO) [37,38]. An arginine residue (R) is added to the oxidized cysteine (C*) by arginyl t-RNA transferases (ATE1/2), which is recognized and ubiquitinated by an E3 ubiquitin ligase, PROTEOLYSIS6 (PRT6) [1,36]. The ubiquitinated ERF-VII proteins are targeted for proteasomal degradation. Under oxygen deprivation (hypoxia), oxidation of cysteine by PCO is inhibited, resulting in the escape of ERF-VII proteins from targeted proteolysis and activation of hypoxia-responsive genes. Alternatively, at least one ERF-VII protein, RAP2.12, physically interacts with plasma membrane-localized acyl-CoA-binding proteins (ACBPs) in an oxygen-dependent manner, limiting its turnover via NERP and participation in the transcriptional activation under normoxia [30]. Under hypoxia, RAP2.12 protein is relocated to the nucleus, activating gene expression [39].

Two independent studies have demonstrated that the N-end rule pathway of targeted proteolysis regulates the turnover of *Arabidopsis* ERF-VII proteins in an oxygen-dependent manner (Figure 3) [30,34]. ERF-VII proteins contain a conserved motif at their amino terminus starting with methionine-cysteine [25], which is widely conserved in higher plants [30,35]. The N-terminal methionine of ERF-VIIs is constitutively removed by methionine aminopeptidases so that the second amino acid, cysteine, is exposed [36]. In the presence of oxygen and nitric oxide, the exposed cysteine is oxidized by plant cysteine oxidases (PCOs) to produce cysteine sulfinic or cysteine sulfonic acid [37,38]. The oxidized cysteine is subsequently conjugated with an arginine residue by arginyl tRNA transferases (ATEs), which is recognized and ubiquitinated by an E3 ubiquitin ligase, PROTEOLYSIS6 (PRT6), and then degraded by the 26S proteasome [1,36]. Because molecular oxygen is a co-substrate of PCO, oxidization of cysteine is restricted under oxygen deprivation. Thus, hypoxia inhibits the oxygen-dependent branch of the N-end rule pathway, leading to the escape of ERF-VII proteins from targeted proteolysis and the activation of ERF-VII-mediated acclimation responses to the stress. 

Besides the N-end rule pathway, the participation of ERF-VII proteins in transcriptional activation is controlled via sequestration of the transcription factor to the plasma membrane (Figure 3) [30]. Under aerobic conditions, RAP2.12 protein interacts with plasma membrane-localized acyl-CoA-binding proteins, ACBP1 and ACBP2. Translocation analysis of photoconverted RAP2.12:mEos protein suggested that oxygen deprivation promotes relocalization of RAP2.12 from the plasma membrane to the nucleus [39]. This mechanism can allow the protection of RAP2.12 against the N-end rule-mediated proteolysis under normoxia, which could enable the immediate activation of acclimation responses when an internal oxygen concentration reached to a critical level.

In *Arabidopsis*, double-knockout mutants of genes encoding arginine transferases, *ate1ate2*, and a single knockout mutant of an E3 ubiquitin ligase, *prt6*, displayed enhanced survival under low oxygen, submergence, and prolonged darkness [34,40]. In addition, the key regulator for submergence tolerance in rice, SUB1A, is not an N-end rule substrate based on *in vitro* data [34]. These results suggest that stabilization of ERF-VII proteins via genetic manipulation of the N-end rule components may result in the improvement of flooding tolerance in plants. In barley (*Hordeum vulgare*), reduced accumulation of *PRT6* transcript by RNAi technology triggered expression of hypoxia-responsive genes and retained chlorophyll degradation under root waterlogging, resulting in the enhancement of biomass production and grain yields as compared to wild type plants [41]. When exposed to submergence and hypoxia, oxygen uptake is severely restricted in the entire plant, whereas shoot tissues still have access to oxygen under waterlogged conditions. It is likely that downregulation of *PRT6* enabled the stabilization of ERF-VII proteins even in the presence of oxygen in shoots of the transgenic plants under waterlogging, contributing to the activation of transcriptional and physiological acclimation in aerial tissues. In wild type plants, however, ERF-VIIs may not be involved in the acclimation to waterlogging in shoot tissues because the targeted proteolysis can degrade these proteins in aerial tissues. Nevertheless, stress-inducible and tissue-specific regulation of the N-end rule components must be an effective approach to enhance tolerance to submergence, waterlogging, and their related stresses without adverse effects on other agronomic traits.

## 3. Submergence, Reoxygenation, and Dehydration

The quiescence survival strategy is successful when submergence water subsides within 14–16 days and plants gain access to the resources (O_2_, CO_2_, light, and nutrients) sufficient to recommence photosynthesis, aerobic respiration, and other metabolic activities. However, re-aeration induces other environmental stresses in plants. For example, sudden exposure to atmospheric oxygen results in oxidative injury [42,43,44]. Reoxygenation stress also triggers a significant drop in hydraulic conductivity in shoots, causing leaf desiccation even in the presence of sufficient soil water [8,10]. The degree of post-submergence stresses depends on the duration of submergence. Reoxygenation following seven days of submergence induced irreversible cellular damage in rice leaves, but desubmergence from three days of inundation did not affect ROS accumulation and lipid peroxidation, resulting in quick recovery from the stress [8,45].

Interestingly, the key regulator of submergence tolerance, *SUB1A*, is involved in the adaptation to post-submergence stresses in rice. Evaluation of genotypes with or without *SUB1A* revealed that *SUB1A* enhances recovery from dehydration through enhanced responsiveness to abscisic acid (ABA), elevated accumulation of mRNAs associated with acclimation to dehydration, and reduction of leaf water loss and lipid peroxidation [8]. Similarly, in the same study, *SUB1A* augmented the abundance of gene transcripts encoding ROS scavengers, limiting accumulation of ROS in aerial tissues and enhancing tolerance to oxidative stress. *SUB1A* also contributed to the maintenance of non-photochemical quenching immediately following de-submergence [45]. Such a *SUB1A*-mediated mechanism can provide protection against sudden exposure to higher light upon reoxygenation, promoting photosynthetic recovery from submergence.

A recent study has revealed that another *ERF-VII* gene, *EREBP1*, is associated with the adaptation to both submergence and drought in rice [46]. Constitutive expression of *EREBP1* elevated mRNA accumulation of genes associated with ABA biosynthesis and the content of ABA in leaves even under non-stressed conditions. Genotypes with overexpressed *EREBP1* displayed enhanced recovery from submergence stress, with restricted underwater elongation and ROS accumulation. These transgenic plants were more vigorously recovered from drought at vegetative and reproductive stages as compared with wild type plants, presumably due to upregulation of drought-responsive genes and increased accumulation of ABA.

Additional evidence for the involvement of *ERF-VII*s in the tolerance to post-submergence-related stresses has been reported in *Arabidopsis*. Accumulation of *HRE2* mRNA was upregulated by osmotic and oxidative stress in *Arabidopsis* seedlings [31]. Overexpression of *HRE2* enhanced tolerance to osmotic and oxidative stresses, whereas osmotic stress induced hyperaccumulation of superoxide anion in *hre2* mutant leaves, reducing seedling survival under the stress. Likewise, inducible expression of each of *RAP2.2*, *RAP2.3*, and *RAP2.12* conferred tolerance to oxidative and osmotic stresses through increased responsiveness to ABA and activation of a subset of dehydration-responsive genes [27]. Based on these results in rice and *Arabidopsis*, it is anticipated that members of *ERF-VII* genes play a prominent role for acclimation to multiple environmental stresses that occur during submergence and following desubmergence. In addition to ERF-VII transcription factors, a sunflower (*Helianthus annuus*) WRKY transcription factor, *HaWRKY76*, functions as a positive regulator for tolerance to submergence and drought [47]. The level of *HaWRKY76* transcript was elevated in response to drought and re-aeration following submergence in leaves of sunflower. Overexpression of *HaWRKY76* in *Arabidopsis* contributed to the conservation of carbohydrate reserves during submergence and the suppression of ROS accumulation following desubmergence, resulting in higher seed production. Under water deficit conditions, the transgenic plants maintained more water in leaves and produced more seeds than wild type. 

## 4. Submergence and Starvation

Due to limited availability of oxygen and light under water, aerobic respiration and photosynthesis are severely restricted in submerged plants. It has been shown that many terrestrial wetland plants form gas films on the super-hydrophobic leaf surface under submergence, facilitating exchange of O_2_ and CO_2_ with surrounding water [48]. In rice, this mechanism enables the maintenance of underwater photosynthesis for 4–5 days [49]. However, prolonged submergence decreases the thickness of gas films, which declines gas exchange and net photosynthesis. Comparative analysis of near-isogenic lines demonstrated that *SUB1A* is not involved in the regulation of underwater photosynthesis in rice. Rather, it appears that *SUB1A* contributes to rapid recovery of photosynthesis following desubmergence [45]. Carbohydrate starvation and an energy crisis are major issues that impact plant growth and survival during submergence. Indeed, the conservation of carbohydrate reserves during submergence is positively correlated with the degree of submergence tolerance [50]. In rice, *SUB1A* economizes carbohydrate reserves in aerial tissue under submerged conditions through suppressed accumulation of mRNAs encoding sucrose synthases and α-amylases [18]. The ability of *SUB1A* to maintain stored carbohydrates was also confirmed in plants exposed to prolonged darkness [51]. *SUB1A* restrained production of ethylene and responsiveness to methyl jasmonate, key hormones involved in the onset of leaf senescence, causing a marked delay of carbohydrate and chlorophyll breakdown in aerial tissue under constant darkness. Recent characterization of *prt6* mutants in *Arabidopsis* has confirmed the link between tolerance to submergence and prolonged darkness [40]. Loss-of-function mutants of *PRT6*, *greening after extended darkenss1* (*ged1*) and *prt6-1*, preserved starch in leaves of adult plants during submergence, contributing to the enhancement of survival under the stress. It was also shown that both of these mutants are significantly more tolerant to prolonged darkness than wild type plants at the seed germination and seedling stages. These results suggest that stabilization of ERF-VII proteins via inhibition of the N-end rule pathway can lead to the economization of carbohydrate reserves under starvation conditions such as submergence and constant darkness in *Arabidopsis*. 

**Figure 4:**
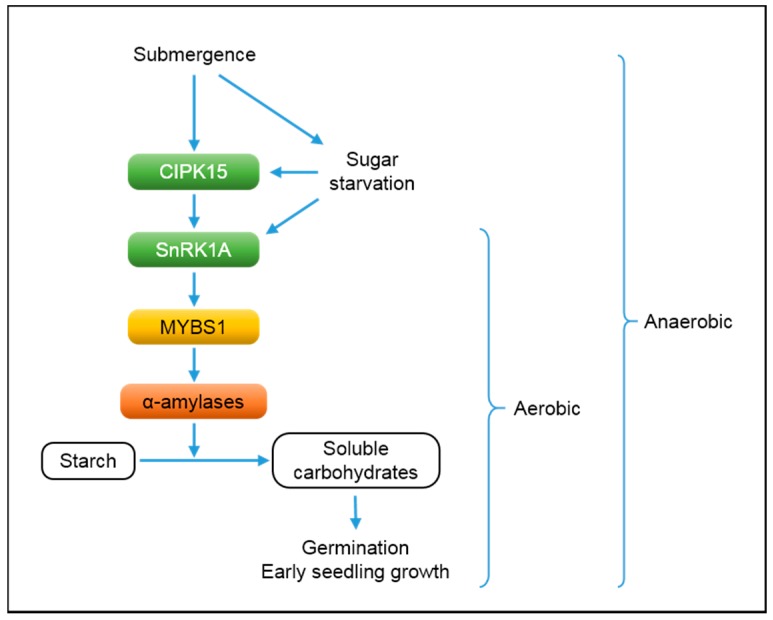
Molecular regulation of germination and early seedling growth in rice under aerobic and anaerobic conditions. Rapid consumption of soluble carbohydrates at the early stage of germination and seedling growth leads to sugar starvation, which stimulates accumulation of an energy sensor protein, SnRK1A [54]. SnRK1A upregulates expression of a MYB transcription factor gene, *MYBS1*. MYBS1 protein directly binds to the promoter region of α-amylase genes, activating the conversion of starch into soluble carbohydrates. Under anaerobic conditions such as submergence, the SnRK1A-mediated signaling cascade is triggered by a calcineurin B-like protein-interacting protein kinase15 (CIPK15) [55]. Physical interaction between CIPK15 and SnRK1 proteins activates the downstream signaling components, promoting starch breakdown to support germination and stand establishment under submergence.

As observed in mature plants of deepwater rice, some non-deepwater accessions can escape from submergence through rapid emergence and elongation of coleoptiles at the seed germination and early seedling stages [52]. This trait is a determinant for successful seedling establishment in direct-seeded systems in areas prone to flooding or where fields are not levelled [53]. It was reported that a carbohydrate starvation/energy depletion sensor, sucrose-nonfermenting1-related protein kinase1A (SnRK1A), plays a critical role in the regulation of seed germination and early seedling growth in rice under both aerobic and anaerobic conditions (Figure 4) [54,55]. SnRK1s are structurally and functionally analogous to their yeast and mammalian orthologs, sucrose nonfermenting1 (SNF1) and adenosine monophosphate-activated protein kinase (AMPK), respectively, all of which are crucial elements for transcriptional, metabolic, and developmental regulation in response to nutrient and energy starvation [56]. At the initial stage of seed germination, soluble carbohydrates are quickly exhausted to support energy demand for repair and synthesis of organelles and other metabolic processes. Sugar starvation promotes protein accumulation of SnRK1A, which stimulates gene expression of *MYBS1* and likely phosphorylates MYBS1 protein [54]. MYBS1 directly up-regulates transcription of α-amylase genes, contributing to the activation of starch breakdown in endosperms. Under oxygen deprivation, a calcineurin B-like protein-interacting protein kinase15 (CIPK15) enhances accumulation of SnRK1A protein and directly interacts with the kinase to trigger the SnRK1A-dependent signaling cascade, promoting anaerobic starch degradation during seed germination and early seedling growth [55]. 

Taking advantage of genetic diversity for the vigor of anaerobic germination within rice accessions, several QTLs affecting survival of submergence at the seedling establishment stage have been identified [57,58]. Of these QTLs, *qAG-9-2* on chromosome 9 was fine-mapped and a trehalose-6-phosphate phosphatase gene, *OsTPP7*, was identified as the genetic determinant on the locus [59]. Introgression of *qAG-9-2* into an intolerant rice variety, IR64, further enhanced mRNA accumulation of submergence-inducible *CIPK15* and *MYBS1* in the tissue containing embryos and coleoptiles, resulting in increased activity of α-amylase and vigorous elongation of coleoptiles under the stress. Trehalose-6-phosphate (T6P) inhibits SnRK1 activity in growing sink organs [60,61,62]. It was proposed in Kretzschmar *et al.* [59] that conversion of T6P to trehalose by TPP7 in local pools may increase sink strength in growing embryos and coleoptiles through activation of the SnRK1-dependent signaling cascade, thereby enhancing starch mobilization and growth vigor during seed germination and early seedling growth under submerged conditions. Genetic and molecular studies have revealed the involvement of SnRK1 and T6P in the regulation of various developmental processes such as seedling growth, root and tuber development, flower initiation, inflorescence branching, pollen development, and embryogenesis in *Arabidopsis*, maize, barley, and potato [60,62,63,64,65,66,67,68,69]. Further studies are required to determine whether the SnRK1 and T6P signaling pathways are associated with the escape response to submergence in other growing tissues such as internodes and petioles. 

## 5. Submergence and Disease

In general, prolonged exposure to abiotic stresses such as drought, salinity, and low temperature attenuates defense responses to pathogens and increases severity of diseases in plants [70,71]. Indeed, a number of studies have demonstrated an antagonistic relationship between ABA-mediated stress signaling and disease resistance at the molecular and physiological levels [72,73,74,75,76,77,78,79,80,81]. These results may reflect a lack of necessity for simultaneous resistance to dehydration and pathogen attack in natural environments because successful pathogen infection requires relatively high humidity [79]. However, other reports identified genes that negatively affect both disease resistance and drought tolerance in rice and tomato (*Solanum lycopersicum*) [82,83], indicating the existence of the pathways co-regulating defense responses to biotic and abiotic stresses. 

In contrast to drought, plants are exposed to high humidity conditions over a flood event, which favor pathogen development and disease transmission [11,12]. At the molecular level, mRNA accumulation of *R* genes is restricted in response to high humidity, resulting in increased susceptibility to *Cladosporium fulvum* in tomato [84,85]. In addition, high humidity suppresses gene expression and kinase activities of mitogen-activated protein kinase3(MPK3) and MPK6 along with reduced accumulation of salicylic acid and hydrogen peroxide, compromising defense responses in lesion-mimic mutants of *Arabidopsis* [86,87,88]. It is expected that submergence and post-submergence injury also impacts plant resistance to pathogens. A recent study has revealed that plants activate defense responses to prepare for the high risk of pathogen infection during and after submergence [89]. Microarray analysis showed that submergence stimulates mRNA accumulation of innate immunity marker genes and WRKY transcription factors in *Arabidopsis* even under pathogen-free conditions. Consistently, pretreatment of *Arabidopsis* seedlings with submergence enhanced resistance to *Pseudomonas syringae* pv. *tomato* in a *WRKY22*-dependent manner under high humidity conditions. The contribution of ERF-VIIs to disease resistance has been evaluated [46,90]. *Arabidopsis* and rice *ERF-VII* genes, *RAP2.2* and *EREBP1*, which are involved in adaptation to submergence, positively regulate disease resistance through increased expression of defense-related genes. Stabilization of ERF-VII proteins via inhibition of the N-end rule pathway under low oxygen may be part of the mechanisms to enhance innate immunity at a high probability of pathogen infection during submergence.

## 6. Submergence and Salinity

Coastal flooding occurs when seawater flows over low-lying areas as a consequence of high tides, storm surges, and tsunami, which can expose plants to submergence and salinity simultaneously. Some halophytes that inhabit coastal regions naturally experience short periods of tidal inundation [91]. Limitation of Na^+^ and Cl^−^ transport into salinity-sensitive tissues/cell-types/organelles and maintenance of local K^+^ concentrations are key factors affecting tolerance to high salt [92,93,94]. When waterlogged with saline water, oxygen deprivation in the root systems restricts energy production required for the regulation of ion transport and homeostasis, leading to increases in Na^+^ and Cl^−^ concentrations and decreases in K^+^ concentrations in shoots [91,95]. It has been recently demonstrated that oxygen availability in root cells is associated with management of cell type-specific ion concentrations in adventitious roots of barley when treated with the combined stresses of salinity and waterlogging [96]. 

The effect of complete submergence on the adjustment of ion transport under high salt conditions is poorly understood. It was suggested in Colmer and Flowers [91] that the inhibition of transpiration under submergence presumably declines the root-to-shoot transport of ions, but salts might be taken up by leaves due to direct contact with saline water. Indeed, when flooded in water containing 25–100 mM NaCl, accumulation of Na^+^ and Cl^−^ in shoots was greater in submerged plants than waterlogged plants in *Melilotus siculus* [97]. Consistently, removal of gas films on the leaf surface elevated the concentrations of Na^+^ and Cl^−^ in shoots under submergence. Prevention of Na^+^ and Cl^−^ transport from roots to shoots is a critical mechanism to protect salt-sensitive leaves when only the root system is inundated in saline water. However, this strategy is unlikely to be beneficial for plants completely submerged in seawater because salt ions can directly enter their leaves. Gas films may serve as an alternative mechanism to hamper the salt entry into leaves in terrestrial wetland plants that occasionally encounter complete submergence in saline water.

Recent molecular analysis suggests that an *Arabidopsis* SnRK1, KIN10, functions as a convergence point that coordinates the antagonistic interactions between salinity and hypoxia tolerance [98]. KIN10 is an upstream component of genes associated with energy starvation induced under darkness, hypoxia, and senescence [67] and positively regulates seedling survival under submergence [99]. AtMYC2 is a transcription factor that activates expression of genes involved in ABA-dependent drought response pathways through direct binding to the ABA-responsive element (*ABRE*) domain [81]. Im *et al.* [98] has demonstrated that phosphorylation of AtMYC2 protein by KIN10 decreases the stability of the transcription activator, resulting in a reduction in *ABRE* promoter activity. Consistently, overexpression of KIN10 reduced tolerance to the combined stresses of submergence and salinity in *Arabidopsis* seedlings. Despite its biological, agricultural, and ecological importance under changing climates, the regulatory mechanism of adaptation to saline submergence has been rarely studied in tolerant species and crops. Comparative genomic and physiological analyses of halophyte and non-halophyte species adapted to wet environments will facilitate the identification of key components and pathways associated with tolerance to the combined stresses.

## 7. Conclusions and Future Perspectives

Plants undergo multiple environmental stresses during submergence and following desubmergence (Figure 1), necessitating the activation of manifold hormonal and signaling pathways coordinating acclimation responses to each challenge. On the basis of genetic and molecular studies, it seems that plant tolerance to submergence and its associated stresses is commonly governed by the core regulatory components encoding transcription factors, protein kinases, and their upstream components (Table 1). For example, rice *ERF-VII* genes, *SUB1A* and *EREBP1* and *Arabidopsis ERF-VII* genes, *HRE1*, *HRE2*, *RAP2.2*, *RAP2.3*, and *RAP2.12* are positive regulators for plant survival under submergence, most of which also confer tolerance to oxidative and osmotic (dehydration) stresses [8,18,19,27,31,32,46]. It is anticipated that ERF-VIIs are generally degraded under oxygen-replete conditions via the N-end rule pathway [30,34]. However, SUB1A protein is not an N-end rule substrate despite containing the conserved N-terminal motif [34]. The escape of SUB1A from the oxygen-dependent proteolysis pathway may enable accumulation of the ERF-VII protein even after reoxygenation, triggering the activation of acclimation responses to post-submergence stresses such as oxidative stress and dehydration. Loss-of-function mutants, *hre2* and *rap2.3rap2.12*, displayed reduced tolerance to osmotic stress in *Arabidopsis* seedlings [27,31]. These results suggest that HRE2, RAP2.3, and RAP2.12 can be accumulated to some extent in wild type plants even under aerobic condition, activating acclimation responses to osmotic stress. Additional regulatory components might be involved in the protection of ERF-VII proteins from the N-end rule pathway under osmotic and other abiotic stresses that can occur following desubmergence. 

Phylogenetic analyses of *SUB1* genes in *O. sativa* suggested that *SUB1A* is a relatively new member of *ERF-VII*s that arose from duplication of *SUB1B* [100], consistent with the observation that *SUB1A* is restricted to a part of *indica* and *aus* accessions [15,17]. Similarly, *SK* genes are also found only in deepwater rice accessions [20]. It appears that extreme responses to submergence (quiescence *vs.* escape) mediated by hormonal regulation have been conferred by these newly formed *ERF-VII* genes. In *Arabidopsis*, all *ERF-VII* genes are associated with tolerance to submergence and oxygen deprivation. It is feasible that natural adaptability to submergence and waterlogging in standard rice accessions are regulated by a common set of *ERF-VII* genes such as *EREBP1*. *SUB1A* and *SK* genes may serve as modifiers of the existing hormonal and signaling pathways, causing intraspecific variation in growth and metabolic responses to excess water.

**Table 1:** Key genes involved in adaptation to submergence and its associated stresses.

Gene	Species	Function	Tolerance	References
*SUB1A*	Rice	ERF-VII TF	Submergence ^a^, oxidative stress ^a^, drought ^a^, prolonged darkness (starvation) ^a^	[8,18,51]
*EREBP1*	Rice	ERF-VII TF	Submergence ^a^, drought ^a^, disease ^a^	[46]
*SNORKEL1/2*	Rice	ERF-VII TF	Submergence (escape response) ^a^	[20]
*RAP2.2*	*Arabidopsis*	ERF-VII TF	Submergence ^a^, low oxygen ^a^, oxidative stress ^a^, osmotic stress ^a^, disease ^a^	[27,29,90]
*RAP2.3*	*Arabidopsis*	ERF-VII TF	Submergence ^a^, low oxygen ^a^, oxidative stress ^a^, osmotic stress ^a^	[27]
*RAP2.12*	*Arabidopsis*	ERF-VII TF	Submergence ^a^, low oxygen ^a^, oxidative stress ^a^, osmotic stress ^a^	[27,30]
*HRE1*	*Arabidopsis*	ERF-VII TF	Submergence ^a^, low oxygen ^a^	[26,32,34]
*HRE2*	*Arabidopsis*	ERF-VII TF	Submergence ^a^, low oxygen ^a^, oxidative stress ^a^, osmotic stress ^a^	[26,31,34]
*PCO1/2*	*Arabidopsis*	Cysteine oxidase	Submergence ^b^	[38]
*ATE1/2*	*Arabidopsis*	Arginine transferase	Low oxygen ^b^	[34]
*PRT6*	*Arabidopsis*	Ubiquitin ligase	Submergence ^b^, low oxygen ^b^, prolonged darkness (starvation) ^b^	[34,40]
*PRT6*	Barley	Ubiquitin ligase	Waterlogging ^b^	[41]
*CIPK15*	Rice	CBL-interacting protein kinase	Submergence (germination and early vegetative stage) ^a^	[55]
*SnRK1A*	Rice	SNF1-related protein kinase	Submergence (germination and early vegetative stage) ^a^	[55]
*KIN10*	*Arabidopsis*	SNF1-related protein kinase	Submergence (early vegetative stage) ^a^, senescence ^a^, salinity ^b^	[67,98,99]
*TPP7*	Rice	T6P phosphatase	Submergence (germination and early vegetative stage) ^a^	[59]
*WRKY76*	Sunflower	WRKY TF	Submergence ^a^, waterlogging ^a^, drought ^a^	[47]
*WRKY22*	*Arabidopsis*	WRKY TF	Disease ^a^	[89]

^a,b^ represent stress tolerance that are positively and negatively regulated, respectively.

It is expected that prolonged submergence leads to nutrient (mineral) deficiency as a consequence of limited transpiration and nutrient uptake (Figure 1). However, the effect of submergence on nutrient deficiency has rarely been studied. It has been recognized that waterlogging reduces soil redox potential, which can increase the availability of Zn, Mn, Fe, and S to the toxic levels [101,102]. Negative impact of oxygen deprivation on root energy supply and membrane integrity can also result in Al, B, and Na toxicity under waterlogged conditions. Under submergence, the influence of nutrient toxicity induced in hypoxic soils might not be critical because limited transpiration severely restricts ion uptake and transport, and toxic ions are considerably diluted with submergence water. Further investigation is required to understand how physical, chemical, and microbial alterations in hypoxic soils affect plant growth, development, and survival under submerged conditions.

Adaptive responses to submergence and its associated stresses are coordinated through synergistic and antagonistic interactions of hormonal, transcriptional, and metabolic pathways [1,16,24,79,103]. It appears that the core genes conferring multiple stress tolerance under submergence and post-submergence act as molecular hubs to connect the signaling cascades regulating individual stress responses. Elucidation of the regulatory mechanisms underlying stability and localization of ERF-VII proteins and their transcriptional activation with partner and effector proteins in different cell types/tissues x stress conditions will facilitate the dissection of the intricate signaling networks governing multiple stress tolerance in plants. 

It has been recognized that plants release a large variety of volatile organic compounds (VOCs) into the surrounding atmosphere in response to various abiotic and biotic stresses such as drought, salinity, low light, high light, oxidative stress, pathogen infection, and insect attack, which function as signaling molecules to trigger acclimation and defense responses to these stresses [104]. Recent studies have suggested that catabolism and degradation of VOCs are associated with plant carbon balance and stress tolerance [105]. Because some of these stresses stimulating emission of VOCs occur during submergence and following desubmergence, these compounds may influence the regulation of adaptation to excess water. It is expected that constitutively and conditionally synthesized VOCs are highly accumulated in submerged tissues due to physical entrapment. Submergence-tolerant species and genotypes may have specific mechanisms to cope with hyperaccumulation of VOCs under water. Future studies will determine the positive and negative effect of submergence-inducible VOCs on adaptation responses and the role of ERF-VIIs in VOC accumulation, catabolism, and degradation under the stress.
